# Speaker-Specific Cues Influence Semantic Disambiguation

**DOI:** 10.1007/s10936-022-09852-0

**Published:** 2022-05-12

**Authors:** Catherine Davies, Vincent Porretta, Kremena Koleva, Ekaterini Klepousniotou

**Affiliations:** 1 School of Languages, Cultures, and Societies, University of Leeds, LS2 9JT Leeds, UK; 2 Department of Linguistics and Languages, McMaster University, Hamilton, Ontario Canada; 3 School of Psychology, University of Leeds, Leeds, UK

**Keywords:** Speaker specificity, Semantic ambiguity, Polysemy, Spoken word recognition, Visual world eye tracking

## Abstract

Addressees use information from specific speakers’ previous discourse to make predictions about incoming linguistic material and to restrict the choice of potential interpretations. In this way, speaker specificity has been shown to be an influential factor in language processing across several domains e.g., spoken word recognition, sentence processing, and pragmatics. However, its influence on semantic disambiguation has received little attention to date. Using an exposure-test design and visual world eye tracking, we examined the effect of speaker-specific literal vs. nonliteral style on the disambiguation of metaphorical polysemes such as ‘fork’, ‘head’, and ‘mouse’. Eye movement data revealed that when interpreting polysemous words with a literal and a nonliteral meaning, addressees showed a late-stage preference for the literal meaning in response to a nonliteral speaker. We interpret this as reflecting an indeterminacy in the intended meaning in this condition, as well as the influence of meaning dominance cues at later stages of processing. Response data revealed that addressees then ultimately resolved to the literal target in 90% of trials. These results suggest that addressees consider a range of senses in the earlier stages of processing, and that speaker style is a contextual determinant in semantic processing.

## Introduction

If a speaker says ‘Bob is a star’, she is using polysemy to communicate that Bob in some way resembles one of the senses of the word ‘star’, for example that he is bright or high-achieving. Her addressee interprets her intended meaning as referring to a human rather than to a celestial body, thereby overriding the dominant sense of ‘star’. Because polysemes (i.e., words with multiple related senses) such as ‘pig’, ‘face’, or ‘fork’ present a range of meaning options, addressees benefit from contextual guidance towards a speaker’s intended meaning. Prior sentential context is a common means of steering interpretation towards a congruous option. For example, in a conversation about traffic directions which mentions ‘fork’, the fork-in-the-road sense will be preferred over the cutlery sense. However, in neutral contexts such as ‘here’s a fork’, addressees must use other cues to disambiguate the intended meaning, such as the relative frequency or dominance of the meanings on offer (for reviews see Rodd 2018; Vitello & Rodd, 2015).

In addition to congruence and dominance, addressees can use extralinguistic cues such as a speaker’s background to disambiguate polysemes. For example, a child is more likely to talk about a slide in a playground than a slide in a presentation. However, the interaction between linguistic and extralinguistic cues has yet to be fully addressed in the experimental literature. In this study, we investigate how a specific aspect of the nonlinguistic context, i.e., *speaker style*, influences the disambiguation of polysemes. We ask how a speaker’s demonstrated style modulates meaning access, with the overarching aim of examining speaker-specificity as an instance of context dependence in language processing. Specifically, we test whether a particular speaker’s style, as established via prior discourse, influences an addressee’s interpretation of a polyseme. We extend the literature on partner-specific effects to polyseme processing, here by manipulating literal vs. nonliteral styles. Together, our empirical outcomes inform the speaker-model account of semantic disambiguation proposed by Cai et al., (2017). This claims that listeners use a variety of linguistic and extralinguistic cues to build up a representation of the speaker, containing information about their profile, such as age, social background, or in our case, speech style. Our findings also inform the literature on polyseme processing; what is the timecourse of speaker-specific effects, and do they influence ultimate reference resolution?

The notion of speakers-as-context is foundational in sociolinguistics (Eckert, 2000; Labov, 2008), which widely attests that aspects of the speaker are themselves valuable sources of information. Broadly construed, addressees use information from speakers’ previous discourse to make predictions about incoming linguistic material and to restrict the choice of potential interpretations of an underspecified signal. Using paradigms that train an association between a specific speaker and their particular way of speaking, such as a novel accent, addressees have been observed to readily use this association to guide their online language processing. Although speaker effects have also been investigated in various psycholinguistic domains, showing that addressees can adapt to different speaker characteristics (Arnold, Hudson-Kam, & Tanenhaus, 2007; Clarke & Garrett 2004; Creel et al., 2008; Kamide, 2012; Maye et al., 2008; Roettger & Franke, 2019; Trude & Brown-Schmidt, 2012), speaker effects on semantic disambiguation have only in recent years started to be investigated (e.g., Bergen & Grodner 2012; Cai et al., 2017; Pogue et al., 2016; Yildirim et al., 2016).

More specifically, speaker identity (also known as speaker- or talker-specificity) has been shown to be an influential factor in language processing across multiple linguistic domains, such as phonology (Roettger & Franke, 2019; Trude & Brown-Schmidt, 2012), spoken word recognition (Arnold et al., 2007; Creel et al., 2008), sentence processing (Kamide, 2012), and semantics/pragmatics (Bergen & Grodner, 2012; Gardner et al., 2021; Pogue et al., 2016; Yildirim et al., 2016).

In spoken word recognition, listeners normalise the variable speech parameters they encounter from different speakers, for example voice onset time or vowel realisation. For example, they use their knowledge of how a particular speaker with a novel accent would pronounce a particular word to rule out or include phonological cohort competitors (Trude & Brown-Schmidt, 2012). Relatedly, speech perception can be influenced by a speaker’s social background, including their expected dialect area (Niedzielski, 1999), where cues to a particular accent or region can influence the perception of phonemes (Hay et al., 2006; Hay & Drager, 2010).

Speaker-specific effects have also been found in the disambiguation of temporarily ambiguous words. For example, addressees look to a particular referent (e.g., a cow vs. a couch) based on a speaker’s prior tendency to refer to one of these referents (Creel et al., 2008). This work builds on earlier research into referential pacts showing that addressees expect their interlocutors to continue referring to objects using a tacitly agreed expression (e.g., ‘the silver pipe’) and will be delayed in resolving reference if that speaker switches to using a different term (e.g., ‘the shiny cylinder’) (Brennan & Clark, 1996; Metzing & Brennan, 2003). The same-speaker advantage has been replicated across a range of studies, typically involving addressees who interact with two different speakers, establishing different referential histories with each Barr & Keysar, 2002; Kronmüller & Barr, 2007; Brown-Schmidt, 2009; Horten & Slaten, 2012; see Kronmüller & Barr 2015 for a meta-analysis). Note that methodological controls used in these studies allow the link between speaker and referential choice to remain arbitrary, i.e., arising only from exposure during the experiment rather than from expectations based on social groups (though see Katz & Pexman 1997; Katz et al., 2004; Staum Casasanto, 2008; Van Berkum et al., 2008; and Van den Brink et al., 2012 for evidence that stereotypical expectations of a particular category of speaker influence linguistic interpretation).

In sentence comprehension, addressees disambiguate syntactic structures based on previously modelled speaker-specific attachment preferences. Kamide (2012) demonstrated that participants learned to anticipate either high- or low attached relative clauses based on prior attachment preferences by particular speakers, and used that knowledge to guide their online processing of temporarily ambiguous structures. A similar effect has also been found by Fine et al., (2013), who showed that comprehenders rapidly adapt to the syntactic statistics of novel linguistic environments.

In pragmatic processing, where speaker meaning is underdetermined by the semantic or sentence meaning, partner-specific stored information may be especially helpful in disambiguating intended meaning. Theoretically, the semantics-pragmatics distinction is characterised as an opposition between *sentence* meaning and *speaker* meaning (Grice, 1975, 1989; Levinson, 1983). Whereas sentence meaning is the abstract, linguistically encoded, literal meaning of a sentence, speaker meaning is the enriched, contextualised, intended meaning as uttered by a speaker^1^. By definition, pragmatic processing is strongly bound to the context in which the linguistic input occurs. In comparison with physical or visual aspects of context, *people* may be particularly potent sources of contextual constraint. Research on a range of linguistic-pragmatic phenomena has demonstrated speaker-specific effects on the interpretation of pragmatic intent. For example, contrastive inferences are suspended if a particular speaker habitually overmodifies a noun, e.g., ‘the tall cup’ when referring to a singleton item (Gardner et al., 2021; Grodner & Sedivy, 2011), and listeners infer that previously under-informative speakers will remain so in their descriptions using different adjectives (Pogue et al., 2016). Similarly, addressees adapt to speaker-specific tendencies in the intended meaning of scalar quantifiers such as ‘some’ and ‘many’ (Yildirim et al., 2016). Such evidence from the experimental pragmatics literature reinforces the effect that speakers have on addressees’ interpretations, over and above linguistic meaning.

The study of the effects of speaker identity on semantic processing is in its infancy. Building on work finding that addressees’ recent and long-term experience with particular words biases disambiguation (Rodd et al., 2013, 2016; Cai et al., 2017) demonstrated that speaker-specific accent cues modulate access to word meaning. Using words that have different meanings in British and American English, listeners were more likely (in word association) and quicker (in semantic relatedness judgement and sentence interpretation) to retrieve an accent-congruent meaning than an accent-incongruent meaning for an ambiguous word. For example, ‘bonnet’ was more readily interpreted as referring to a car part (rather than to a hat) when it was spoken in a British than in an American accent. These results are taken to support a speaker model account of semantic processing, in which comprehenders determine the characteristics of their interlocutor and use this knowledge to guide meaning retrieval, in a route parallel to but separate from the individual lexical representations.

The current experiment manipulates speaker style. To our knowledge, this is the first experiment to investigate its effects on semantic disambiguation. In an exposure phase, we trained participants to learn whether a particular speaker characteristically uses nonliteral expressions such as metaphors and idioms in their speech, or not^2^. Then in a test phase, participants followed the instructions of the same on-screen speakers to find visual objects. If an addressee has established a model of their interlocutor as someone who uses a characteristic speech style (literal vs. nonliteral), they might have expectations about the kind of constructions that particular speaker will use, and what meanings they intend these constructions to have. As the literature on speaker-specific effects has established, knowledge associated with a specific speaker prompts expectations about the content of incoming messages. Polysemous words (especially those whose senses are motivated by metaphors) provide a novel yet highly appropriate testing ground for speaker-specific effects on semantic processing. Evidence suggests that multiple senses of polysemes are available during processing potentially via a core meaning representation or an underspecified representation model (Ferreira et al., 2002; Ferreira & Patson, 2007; Frisson, 2009; Frisson & Pickering, 1999; Klepousniotou et al., 2008). Polysemy also provides a neat parallelism between alternative senses (literal; nonliteral) and our predictor variable, i.e., speaker style (literal; nonliteral).

To our knowledge, this is the first experiment to investigate the psycholinguistic effects of literal vs. nonliteral speech style. However, a small body of comparable literature has investigated pragmatic processing as a result of speakers who have been established as having a characteristic speech style, i.e., whether they are typically sarcastic or not. Regel et al., (2010) established a speaker as sarcastic, then found larger ERP amplitudes (P200 and P600) when participants heard sarcastic comments (relative to literal comments) from the non-sarcastic speaker, whereas the amplitudes were equal for literal and sarcastic comments made by the sarcastic speaker. This provides evidence for online speaker style effects in pragmatic processing, whereby participants were surprised when the literalness of the speaker violated their knowledge of that speaker. In contrast, Giora et al., (2007, expt. 1) and Fein et al., (2015) recorded slower reading times for sarcastic comments (cf. literal comments) even after speakers had been established as typically sarcastic. These two studies used holistic reaction time measures rather than the online analysis used by Regel et al., (2010). Our study therefore uses eye tracking measures to provide the live timecourse of speaker-driven effects on semantic-pragmatic processing. Further, our stimuli are presented naturalistically as a continuous audio stream, unlike all three previous studies in this domain, which used sentence-by-sentence or word-by-word self-paced presentation, which is likely to have prevented participants from engaging naturally with the dialogue.

Turning to the semantic phenomenon that this study uses, polysemy occurs when words have several different yet semantically related interpretations or senses, such as ‘beam’ (structural support vs. ray of light) and ‘University’ (a building hosting research and education vs. its organisation and management). Most words in a language can convey a range of meanings, with over 80% of English words having more than one dictionary entry (Rodd et al., 2002). Polysemous words are intrinsic to the flexibility of language, allowing speakers to capture nuances of meaning without the cost of a lengthy corresponding lexicon. They also enable easy and transparent coinage of labels for new concepts, such as ‘ghosting’ and ‘tablet’. Many studies investigating the processing of polysemes have been concerned with whether it proceeds in a similar way to the processing of homonyms (words with semantically unrelated interpretations like ‘bank’ and ‘pupil’), i.e., whether meaning dominance and discourse context influence the relative activation of the available meanings or senses. Empirical findings have largely converged to show that, in contrast to homonyms, metonymic polysemes (e.g., ‘lamb’ in its animal vs. food senses) do not typically show dominance effects, and no extra processing effort is involved when the subordinate meaning is intended Frazier & Rayner, 1990; Frisson & Pickering, 1999, 2007; McElree et al., 2006; but cf. Klein & Murphy, 2001), while metaphorical polysemes (e.g., ‘rat’ in its animal vs. insult senses) seem to have their subordinate, nonliteral senses mediated through their dominant, literal senses (Klepousniotou et al., 2008). Thus, evidence suggests that no immediate semantic commitment is necessarily made to a specific sense in polyseme processing (Frisson, 2009; Klepousniotou et al., 2008). Furthermore, in contrast to homonyms, all senses of polysemes are available at early stages of processing (Klepousniotou et al., 2012) and show sustained activation at later stages too (MacGregor et al., 2015). The assumption that all senses are potentially available during processing is important for investigating whether speaker identity influences polyseme interpretation. If there was a strong commitment to a single meaning in response to our stimuli, this might mask the (potentially weaker) effect of speaker identity.

Our experiment reveals the incidence and timing of speaker-specific effects on semantic disambiguation during the processing of polysemes, before commitment to a specific interpretation. Besides meaning dominance, the only biasing context comes from the speaker themselves; there is no prior sentential or discourse context weighting one interpretation over another. The visual world paradigm allows us to examine any such speaker bias from the earliest moments of processing, up to the moment that a specific sense is selected.

Our primary research question asks whether speaker-specific style influences semantic disambiguation. When participants are trained to associate a particular speaker with a particular style, does their model of the speaker bias them to a particular interpretation of a polysemous word? The styles used were nonliteral (i.e., a speaker who used many idioms, metaphors, similes) and literal (i.e., a speaker who did not use any of these forms). We hypothesised that after habituation, when interpreting polysemous words with a literal and a nonliteral meaning, addressees would experience more interference from a nonliteral speaker than from a literal speaker before resolving to the literal target with their mouse-click response. We expected that the ambiguity would ultimately be resolved to the literal target due to meaning dominance effects. Interference was indexed by (a) *longer reaction times* for resolution to the literal target in the nonliteral style condition, and (b) *a lower proportion of looks to the literal target* while processing the ambiguous noun in the nonliteral style condition. Such patterns would reflect addressees’ assumptions that the nonliteral speaker may have intended their referring expression to have a nonliteral meaning.

## Method

### Participants

33 participants were recruited from the student community at the lead author’s University. All were native speakers of British English, right-handed, and had normal or corrected-to-normal vision and hearing. Each participated voluntarily. Four participants were excluded due to technical malfunction, leaving the remaining sample at n = 29 (11 males) with a mean age of 23 years (range 18–35; *SD =* 5). The study was approved by the Faculty research ethics committee at the lead author’s institution.

## Design and Materials

The experiment consisted of an exposure phase followed by a test phase. The aim of the exposure phase was to establish an association between the speaker and their speech style (literal or nonliteral). The aim of the test phase was to measure whether this association influenced participants’ interpretation of polysemous words. Speakers were differentiated by gender (following Kamide 2012), counterbalanced between style conditions.

### Exposure Phase

Six narratives were created on three different themes (anger; sadness; fear). Three of the narratives were nonliteral and each contained 14 nonliteral expressions. The other three narratives were matched for theme but included only the corresponding literal expressions (see Table 1 for an example narrative pair). Narrative pairs were matched for length (mean word count 147, *SD =* 8), propositional content, and syntactic complexity. One male and one female actor were recruited to present the narratives. The actors were matched on approximate age and ethnicity, and both spoke with a Standard British English accent. They received a small payment for their time. They learned the narratives verbatim and were video recorded speaking directly to a camera in front of a neutral background, framed to the head and shoulders, using a natural delivery with a consistent and matched speed. Each spoken narrative was approximately 50 s in duration.

**Table 1:** Example narrative pair used in the exposure phase, presented auditorily

Nonliteral	Literal
Mrs. Simmons is a **bear** about cleanliness.	Mrs. Simmons loves cleanliness.
Her house is always very clean. She is always **prowling around** with a duster in hand	Her house is always very clean. She is always walking around with a duster in hand
and attacks every speck of dust like a personal enemy.	and attends to every spot of dust she sees anywhere in the house.
It’s not easy on her kids. She watches them like a **hawk** to see whether they make a mess.	It’s not easy on her kids. She watches them very closely to see whether they make a mess.
She is not really a **hot-headed** person	She does not lose her temper quickly
but she will always be **simmering with anger** when cleaning the house.	but her anger will gradually increase when cleaning the house.
She **gets all steamed up** when her family does not cooperate.	She gets angry when her family does not cooperate.
The **pressure really builds** when she is cleaning her kids’ room.	The pressure increases when she is cleaning her kids’ room.
If they walk in with muddy shoes, **she blows her top.**	If they walk in with muddy shoes, she immediately starts shouting at them.
Mr. Simmons does not say much when his wife **unleashes her anger;**	Mr. Simmons does not say much when his wife starts shouting;
he gives her one **poisonous** look	he gives her a very unfriendly look
and continues to **nourish his resentment.**	and continues to feel resentment towards her.
He finds her **caustic anger** almost unbearable at times	He finds her excessive anger almost unbearable at times
but since he does not want to confront her openly, they live in a constant **bitter concoction of anger and resentment.**	but since he does not want to confront her openly, they live a life full of anger and resentment.

Speaker style, gender, and presentation order were counterbalanced over four lists. List 1 featured the male speaker presenting the literal narratives and the female speaker presenting the nonliteral narratives (presentation order was reversed in list 2), and list 3 featured the male speaker presenting the nonliteral narratives and the female speaker presenting the literal narratives (presentation order was reversed in list 4). To monitor whether the participants had associated speaker with style after the exposure phase, we created six pairs of shorter written vignettes on the same themes as the exposure narratives; each pair was matched for word count (*M =* 31, *SD =* 6) across all six vignettes. Four of these vignettes had been redacted from the narratives shown in the exposure phase, and two were novel. Example vignette pair used in the exposure phase, presented visually.

A. *It was a stormy meeting. Bill’s voice was boiling with anger; he refused to take responsibility. Everyone was getting hot under the collar. When he asked for Tim’s resignation, I flipped my lid.*
B. *It was a difficult meeting. Bill’s voice showed his anger; he refused to take responsibility. Everyone was getting annoyed. When he asked for Tim’s resignation, I lost control.*

See *Procedure* for details of how these extra vignettes were used to check the association between speaker and style.

### Test Phase

**Fig. 1:**
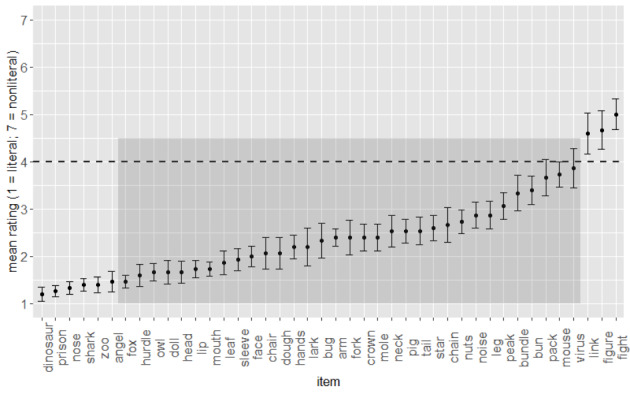
Mean literality ratings for all pretested stimuli. Dashed line shows rating scale midpoint (‘neither literal nor nonliteral’). Shaded box indicates the 32 items taken forward as experimental stimuli. Error bars = SE

A list of 42 metaphorically polysemous words, e.g., arm, fork, crown, was constructed from stimuli previously used by Klepousniotou et al., (2012), and MacGregor et al., (2015). Visual representations of the literal and nonliteral interpretation of each word were sourced from Google Images. The words were then pretested for literal or nonliteral bias. Fifteen independent raters, all speakers of British English and with at least 14 years of full-time education were recruited via Prolific Academic. They rated the 42 preselected written words for literal / nonliteral bias using a 7-point Likert scale with the literal meaning depicted at one end of the scale and the nonliteral meaning at the other, counterbalanced. For example, for the word ‘face’, a picture of a human face appeared at one end of the scale and a clock face at the other. On the scale provided, one endpoint indicated a solely literal meaning, the mid-point indicated a meaning which was equally literal and nonliteral, and the other endpoint indicated a solely nonliteral meaning. Raters’ choice of point on the scale was converted to a numeric score from 1 (solely literal) to 7 (solely nonliteral). By-item mean ratings ranged from 1.2 (dinosaur) to 5.0 (fight). Of the 42 items, 32 were rated between 1.5 and 3.9, i.e., slightly to strongly literal-biasing (M = 2.5; SD = 0.6), while ten were at the extreme ends of the scale. The ten extreme items were removed and the 32 items went forward for use in the test phase. The pretest ensured that the dominant meaning of all of the experimental items was literal. The final list of stimuli is presented with their pretest ratings in Fig. 1.

**Fig. 2:**
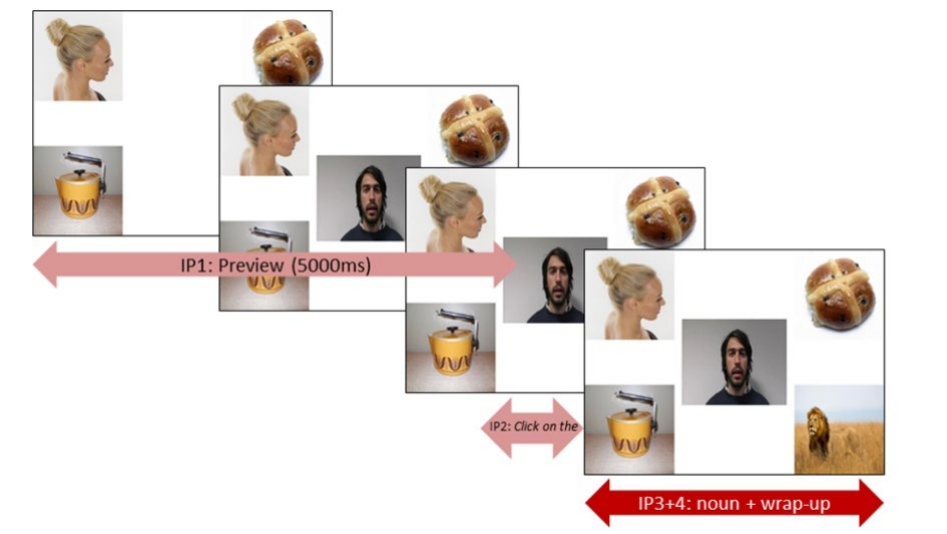
Trial sequence (numbers in this caption refer to the display screens depicted above left-to-right). (1) The image preview was presented for 3500 ms, followed by (2) a preview of the speaker alongside the images for 1500 ms. (3) The speaker then produced the utterance ‘*Click on the bun*’ (≈ 1500 ms), at which point the participant clicked on the image which best matched the referring expression. (4) The speaker and the images stayed on screen for a wrap-up period lasting 2000 ms after the mouseclick. Superimposed arrows show correspondences between display screens and interest periods (IP1-4). Precise durations of each interest period are given in *Materials*

Composite displays for the test items were then created. Each item featured an array of four images; one literal target, one nonliteral target (each taken from the pretest), and two distractors (see Fig. 2 for an example item within its trial sequence). The four images in each composite item were matched for visual complexity and colour palette using Likert-scale ratings from independent, blinded raters (n = 12). The distractors did not share the initial sound or rhyme with the critical word. Each image within the display, including the speaker, measured 300 × 300 pixels, and the on-screen location of each image type was balanced across trials. The actors who had narrated the vignettes in the exposure phase appeared as head-shot videos in the middle of each composite item. These videos showed the speaker instructing the participant to click on one of the images using the simple command ‘*click on the [noun]’.* Four lists of 32 pseudorandomised items were created, with no more than three trials from each of the two speaker style conditions appearing consecutively. Lists were counterbalanced for style and gender, i.e., items 1–16 were produced by the male and 17–32 by the female in list 1, genders reversed in list 2, presentation order block-randomised in lists 3 and 4. All stimuli are available at https://osf.io/q7f2r/.

The test phase was programmed using Experiment Builder v.1.10.1630 (SR Research). We created five visual interest areas (literal target; nonliteral target; distractor 1, distractor 2, other), and four audio interest periods. These were IP1: image / speaker preview (3500 + 1500 = 5000 ms); IP2: utterance onset – noun onset, i.e., *‘click on the’* (variable duration: range 756–1127 ms, *M =* 925 ms, *SD =* 78 ms); IP3: noun onset – mouseclick (variable duration: range 433–6036 ms, *M =* 2143 ms, *SD =* 908 ms); IP4: wrap-up (duration of 2000 ms after mouseclick).

## Procedure

All participants were tested in a single session that lasted approximately 30 min. They were tested individually in a purpose-designed testing suite at the lead author’s University.

After giving their informed consent and receiving instructions, participants began the exposure phase. On a standard PC monitor with external amplification, they watched the three pairs of video narratives produced by the speakers using either a literal or a nonliteral style. Each successive narrative was activated by the participant. They were told to pay close attention to the way that each speaker used language. After this implicit exposure, participants read six short written vignette pairs on screen (a literal and nonliteral variant on the same theme) which were displayed above small headshots of each speaker. On reading each vignette pair, participants were asked to match each vignette to the speaker who was more likely to have produced it. They signalled this link orally to the experimenter, e.g., *‘Story A was by the girl; B was by the guy’.* As a group, the final sample of participants (n = 29) correctly attributed 89% of vignettes to the correct speaker (*SD* = 21), and all participants reported a high degree of confidence in their own selections at the end of the exposure phase. The exposure phase took approximately 10 min to complete. Participants then proceeded to the test phase.

For the test phase, participants were seated in front of a monitor with their eyes approximately 60 cm from the display using a chinrest. We used an SR Research EyeLink 1000Plus eye tracker, sampling at 1000 Hz from the dominant eye. A nine-point calibration and validation was performed. Participants were then instructed to follow the on-screen instructions given by each speaker. Visual stimuli were presented on a Dell 17” flat panel monitor with a content area of 1280 × 1024 pixels visible to the participant. Participants selected the image corresponding to their choice using a mouse.

Four practice trials were presented, followed by the 32 experimental trials (16 featuring each speaker) with a break at the half way point, after which calibration and validation were repeated. The trial procedure is shown in Fig. 2. Between each trial, participants were shown a single centrally-located dot to correct for any drift in the calibration. Trials were automatically terminated seven seconds after the onset of the speaker’s command if no mouse response was made. The mouse cursor returned to the centre of the display at the beginning of each trial. The test phase lasted approximately 10 min. A full debrief ended the procedure.

Three types of analyses were conducted: response type analysis, reaction time analysis and eye tracking analyses. Across all three analyses, Speaker Style was manipulated within participants (literal speaker; nonliteral speaker). Additionally, the reaction time analysis included Response Type as an independent variable (literal target; nonliteral target). Outcome variables were (1) target response, i.e., frequency of clicks to the literal vs. nonliteral target; (2) reaction time between noun onset and click response; (3) visual preference for the literal target within two critical time windows (noun; post-noun).

## Results

### Response Type Analysis

A single trial with a response time of 188 ms after the onset of the noun was excluded from the analysis as it was unlikely to reflect true processing. Thus, the trial with the shortest response time was 432 ms. Additionally, 14 trials that timed out, i.e., did not elicit a mouse click response, were excluded. After these exclusions, 913 trials (98% of the original dataset) went forward for analysis. Of these trials, 824 were resolved to the literal target (90%) and 89 to the nonliteral target (10%).

**Fig. 3:**
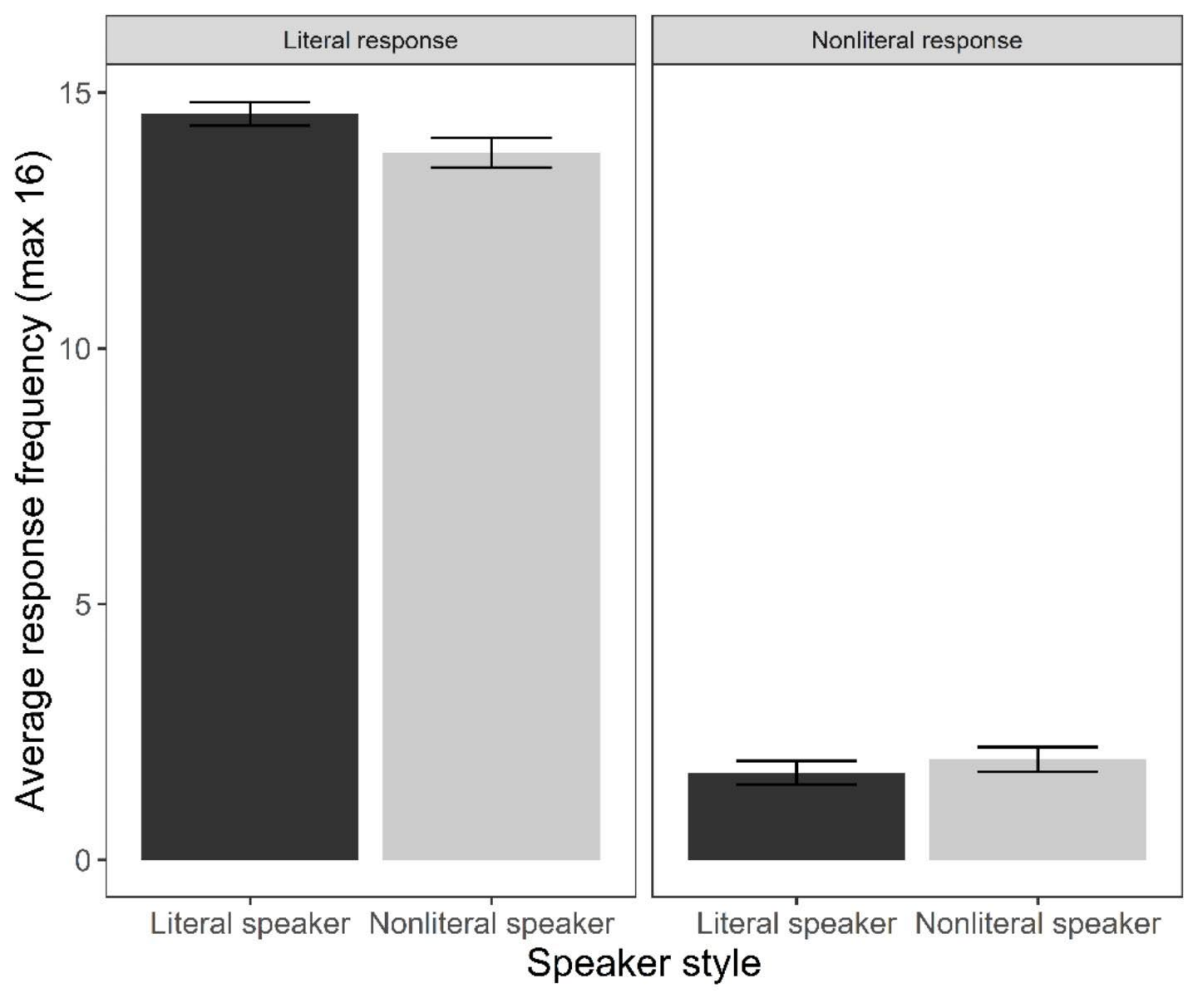
Average response frequency by speaker style and response type. Error bars represent standard error

To examine the effect of speaker style on the probability of eliciting a nonliteral response, a logistic mixed-effects model was fitted to the data and log-likelihood ratio tests were used to assess the contribution of the predictors to the model. In addition to speaker style, trial order (centred) was included as a fixed effect, with random effects for subjects and items. The maximum random structure supported by the data was determined through backward fitting (see Matuschek et al., 2017). This random structure included random intercepts for both subjects and items. The model summary is provided in Table 2. Statistical analysis was performed using mixed-effects regression as implemented in the package *lme4* (Bates et al., 2015) in R (R Core Team, 2018).

**Table 2:** Model summary for effect of speaker style on response type

Random effect	Variance	*SD*		
Item (Intercept)	5.345	2.312		
Subject (Intercept)	0.372	0.610		
**Fixed effect**	**Estimate**	***SE***	***z*** **value**	***p*** **value**
Intercept	-3.968	0.858	-4.625	< 0.0001
Speaker style (nonliteral)	0.948	0.978	0.97	0.332
Trial	-0.029	0.017	-1.723	0.085

As Fig. 3 shows, there was a small numerical preference for the literal target in response to the literal speaker (*M =* 14.6, *SD =* 1.2) compared to the nonliteral speaker (*M =* 13.8, *SD =* 1.6), and a small numerical preference for the nonliteral target in response to the nonliteral speaker (*M =* 1.9, *SD =* 1.3) compared to the literal speaker (*M =* 1.2, *SD =* 1.2). However, speaker style was not a statistically significant predictor of the probability of nonliteral responses. This is in line with our hypothesis that participants would resolve reference to the literal target due to its meaning dominance, regardless of speaker style. There was no effect of trial order, meaning that a particular response (literal; nonliteral) did not become more likely as the experiment proceeded.

### Reaction Time Analysis

Reaction times were measured from the onset of the noun to the point at which the participant clicked on the target image. The mean noun duration was 589 ms (*SD =* 132, range 359–924). The same exclusion criteria as in the response type analysis above were used here. For the reaction time analysis, only trials resolved to the literal target were considered because our research question focuses on the extent of interference while participants resolve to the dominant sense. As stated above, this was 90% of the dataset.

**Fig. 4:**
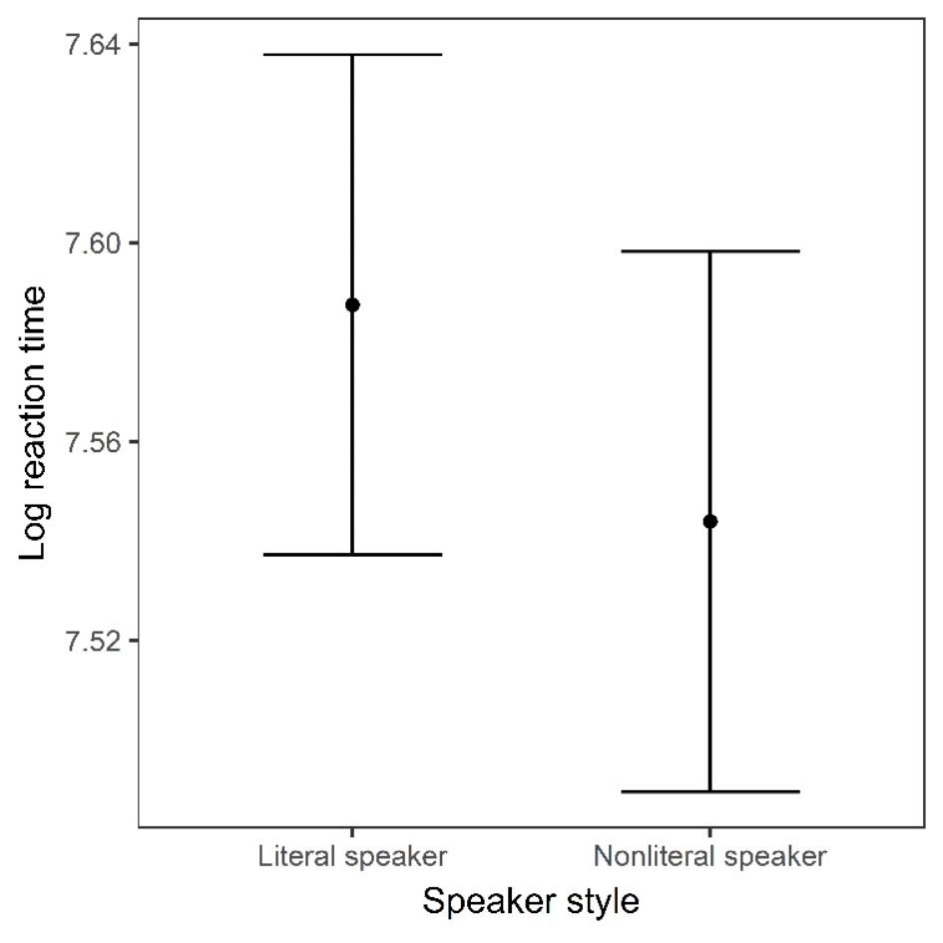
Grand average log-transformed reaction times to literal target from noun onset to mouse click, by speaker style. Error bars represent standard error

Reaction times were log-transformed prior to analysis to remove some of the skewness in the data (Baayen & Milin, 2010). To examine the effect of speaker style on reaction times to the literal target, a linear mixed-effects model was fitted to the data. In addition to speaker style (literal/nonliteral), trial order (centred) was included as a fixed effect, with random effects for subjects and items. Again, the maximum random structure supported by the data was determined through backward fitting. This random structure included random slopes for trial order by subject, and random intercepts for both subjects and items. This model was refitted with data within ± 2.5 standard deviations of the residuals of the model, resulting in the removal of 20 datapoints, i.e., 2.4% of the data (Baayen, 2008). We will refer to this as the final model; the summary can be found in Table 3. The *p*-values reported were obtained using the R package *lmerTest* (Kuznetsova et al., 2015).

**Table 3:** Model summary for effect of speaker style on log-transformed reaction times

Random effect	Variance	*SD*	Corr.		
Item (Intercept)	0.031	0.175			
Subject (Intercept)	0.066	0.257			
Trial (Slope)	0.000	0.006	0.02		
Residual	0.043	0.208			
**Fixed effect**	**Estimate**	***SE***	**df**	***t*** **value**	***p*** **value**
Intercept	7.633	0.067	55.538	113.981	< 0.0001
Speaker style (nonliteral)	-0.018	0.064	29.813	-0.276	0.784
Trial	-0.002	0.002	33.055	-1.579	0.124

As Fig. 4 shows, speaker style did not significantly influence reaction times to resolve the referring expression. There was no effect of trial order. Thus, against our prediction that the nonliteral speaker would trigger interference from the nonliteral target during processing, we did not find a slowdown. Participants resolved to the literal target equally quickly regardless of whether the speaker had used a literal or a nonliteral style during the exposure phase.

### Eye Tracking Analyses

***Data preparation***.

The sample data were exported using Data Viewer v.2.5.0 (SR Research) relative to the onset of the noun. The data were further prepared for analysis and visualised using the *VWPre* package, version 1.1.0 (Porretta et al., 2018) in R. The data were first converted to proportion of samples falling within and outside each of the predefined interest areas in 50 ms windows (50 data points). These proportions were then converted to empirical logits using 50 samples per bin and a constant of 0.5 to yield an unbounded measure (see Barr 2008).

Given the study’s focus on interference effects during resolution to the dominant sense, plus the preponderance of literal target responses (90%), we maintained our approach of analysing data from trials resolving to the literal target only (*n* = 824). For the eye tracking analysis, this also has the advantage of maximising the amount of data during the time windows of interest as nonliteral target responses were relatively late-arriving (mean RT for literal responses = 2073 ms, mean RT for nonliteral responses = 2786 ms; estimate = -0.09, SE = 0.04, *p* < .05).

***Eye movements during test***.

Prior to analysis, inspection of the pre-stimulus interest periods - consisting of the image preview, speaker preview, and carrier phrase (‘*click on the*’) - revealed that all four images received approximately equal proportions of fixations during the image preview and that participants tended to fixate the face during the speaker preview and continued through the carrier phrase. This pattern held across conditions, and suggests that there was no anticipation of a particular referent(s) based on the appearance of a particular speaker during this pre-noun period.

For analysis, the critical interest period ran from noun onset until the trial end (i.e., IP3 and IP4 as described in Methods) which had a mean duration of 4143 ms (SD = 908, range 2433–8036). This ensured that data were present from all participants, as mouse click response times varied. Two time windows were identified for analysis from within the critical interest period: 400–850 ms (‘early’) and 850–1300 ms (‘late’). The first window was selected for two reasons. First, it captures processes associated with hearing the noun which had a mean duration of 589 ms (*SD* = 132). Second, it takes approximately 200 ms to plan and execute a saccade based on an auditory signal (Fischer, 1992; Matin et al., 1993). The second window was chosen to capture processes after the noun had been heard.

**Fig. 5:**
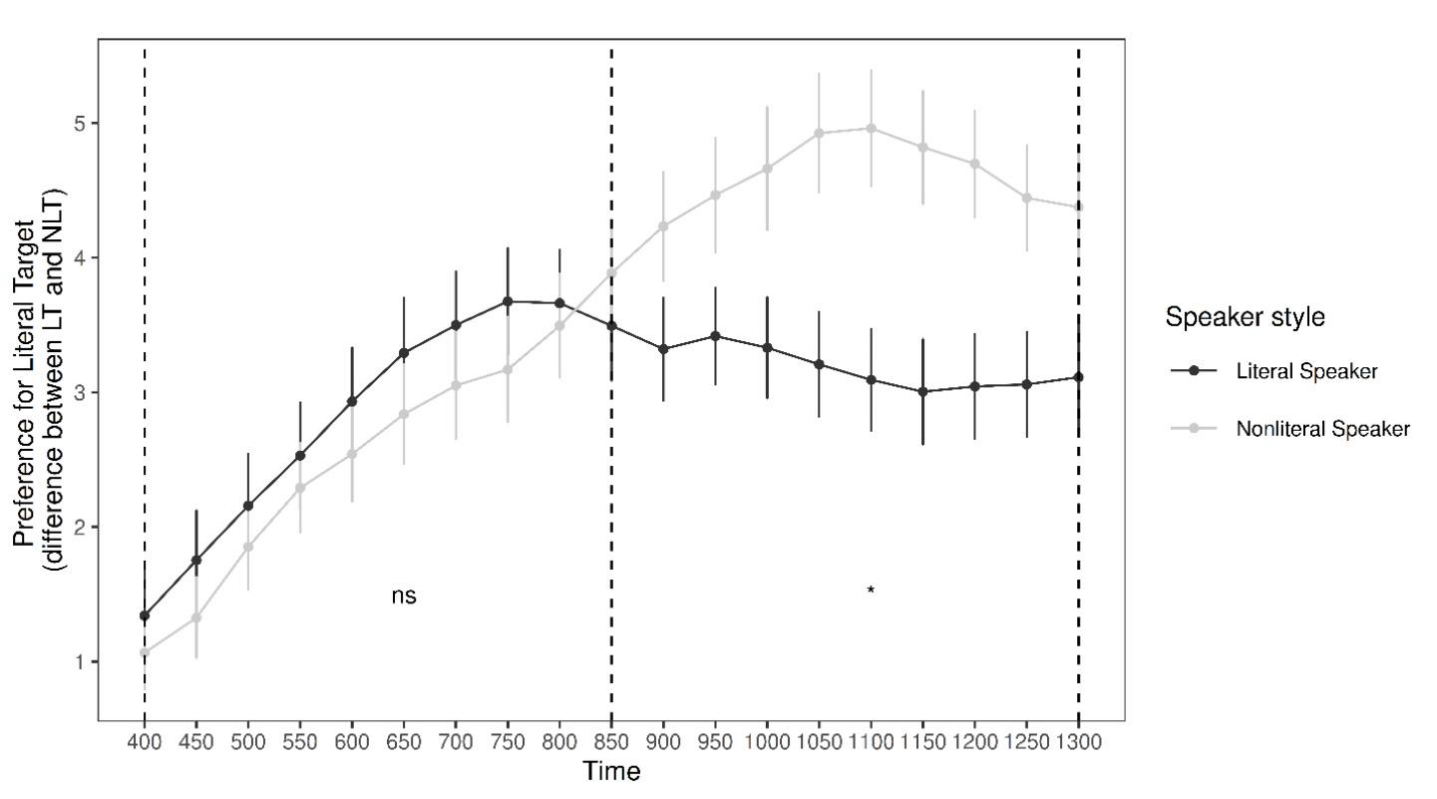
Average difference in looks between the literal target and the nonliteral target by speaker style across the two analysis windows (indicated by dashed vertical lines). Error bars represent standard error

As the research question focuses on the influence of speaker style on preference for the literal target, the difference in looks between the literal target and the nonliteral target was taken as the response variable. This allowed for the examination of the preference for the literal target over the nonliteral target in looking behaviour. These differences, presented in Fig. 5, were averaged by condition within each time window and analysed separately.

***Early window***.

**Fig. 6:**
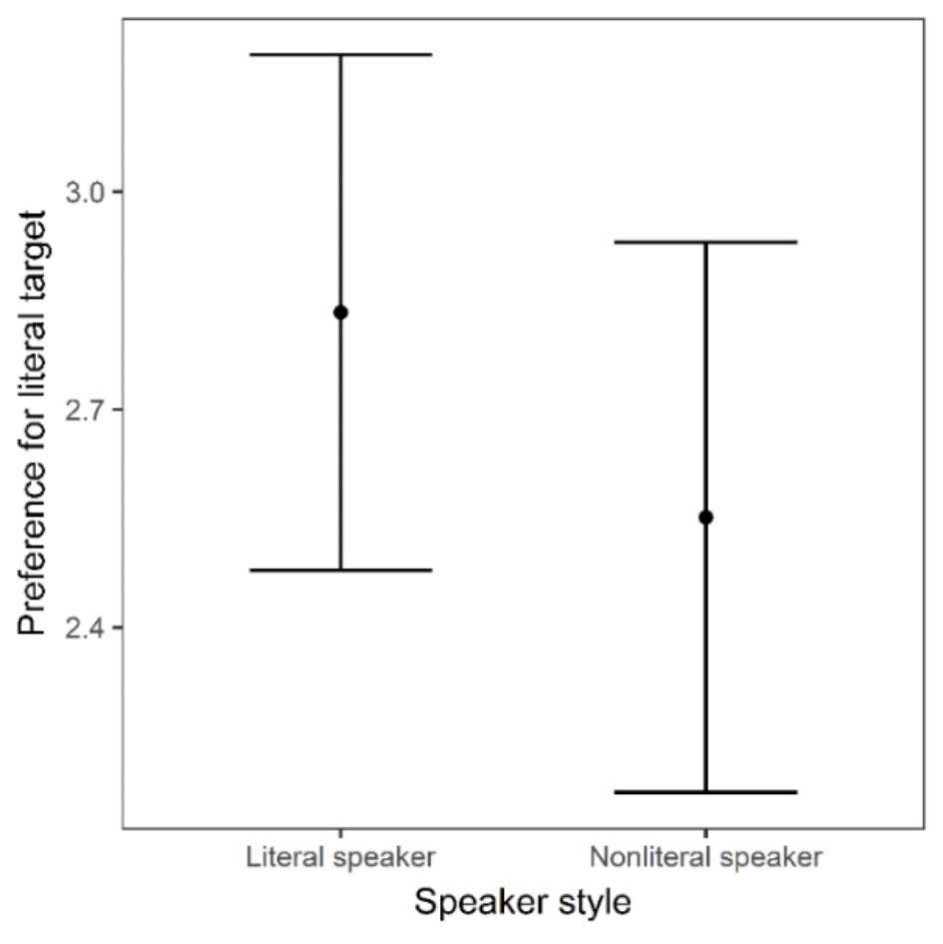
Average difference in looks between the literal target and the nonliteral target by speaker style within the early analysis window (400–850 ms). Error bars represent standard error

A linear mixed-effects model was used to analyse literal target preference (i.e., average difference in looks to the literal target and looks to the nonliteral target, described above) as a function of speaker style between 400 ms and 850 ms (see Fig. 6). The model fitting and trimming procedures were the same as above and the final model included speaker style and trial order (centred) as fixed effects, with random intercepts for subjects and items. A summary of the final model is presented in Table 4.

**Table 4:** Model summary for effect of speaker style on literal target preference during the early time window (400–850 ms)

Random effect	Variance	*SD*			
Item (Intercept)	0.675	0.821			
Subject (Intercept)	3.146	1.774			
Residual	12.903	3.592			
**Fixed effect**	**Estimate**	***SE***	**df**	***t*** **value**	***p*** **value**
Intercept	2.532	0.476	65.003	5.318	< 0.0001
Speaker style (nonliteral)	-0.261	0.388	26.923	-0.671	0.508
Trial	0.034	0.017	655.298	2.044	0.041*

**Fig. 7:**
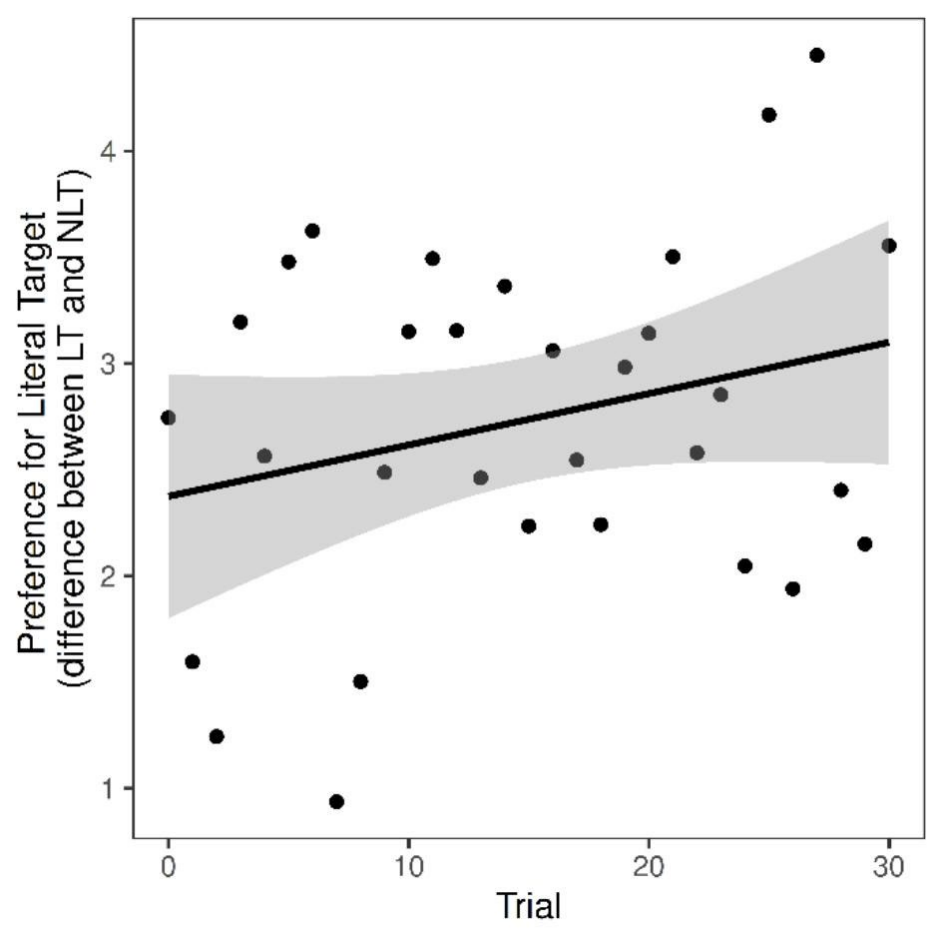
Preference for literal target in the early analysis window, plotted by trial order. Shaded area = SE

As Figs. 5 and 6 show, while there was a trend in the data for a reduction in preference for the literal target in response to the nonliteral speaker as predicted, there was no statistically significant effect of speaker style in the early time window. However, there was a statistically significant effect of trial order, such that preference for the literal target increased in this time window across the experiment (see Fig. 7).

***Late window***.

**Fig. 8:**
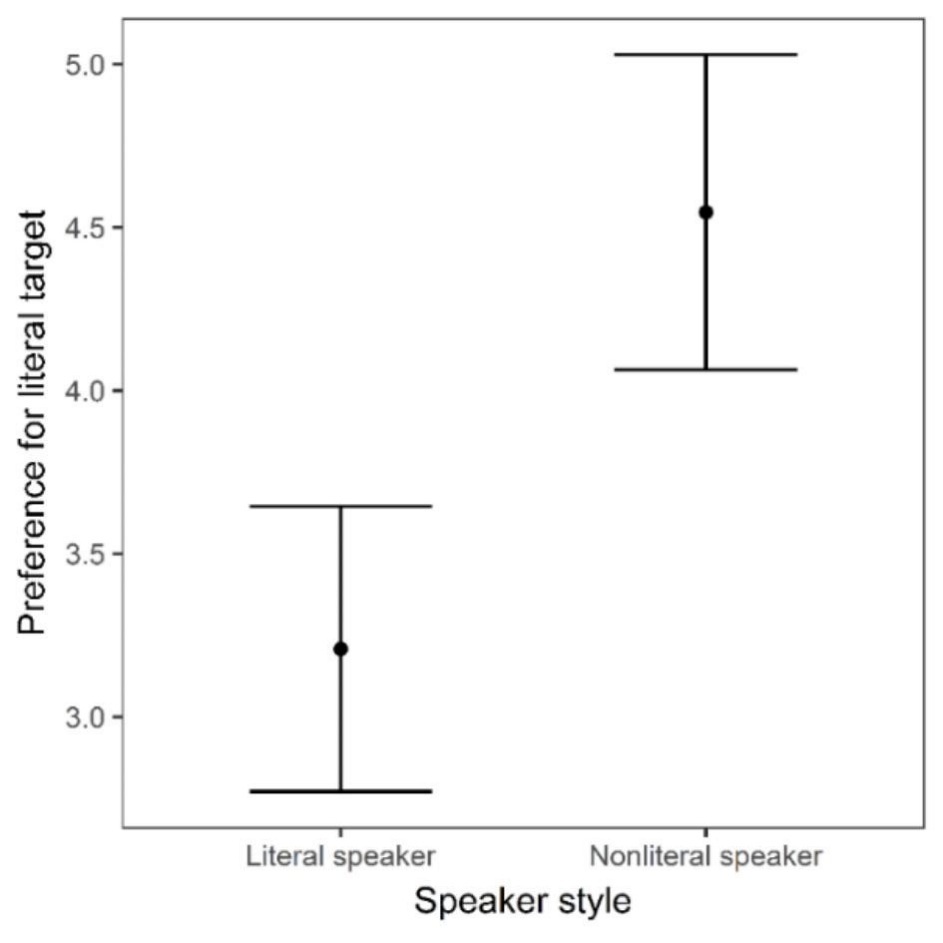
Average difference in looks between the literal target and the nonliteral target by speaker style within the late analysis window (850–1300 ms). Error bars represent standard error

A linear mixed-effects model was used to analyse literal target preference (i.e., average difference in looks to the literal target and looks to the nonliteral target, described above) as a function of speaker style between 850 ms and 1300 ms (see Fig. 8). The model fitting and trimming procedures were the same as above and the final model included speaker style and trial order (centred) as fixed effects, with random intercepts for subjects and items. A summary of the final model is presented in Table 5.

**Table 5:** Model summary for effect of speaker style on literal target preference during the late time window (850–1300 ms)

Random effect	Variance	*SD*			
Item (Intercept)	0.434	0.659			
Subject (Intercept)	4.276	2.068			
Residual	20.637	4.543			
**Fixed effect**	**Estimate**	***SE***	**df**	***t*** **value**	***p*** **value**
Intercept	2.956	0.539	61.042	5.482	< 0.0001
Speaker style (nonliteral)	1.195	0.399	26.246	2.998	0.005**
Trial	0.030	0.020	505.480	1.495	0.136

As Figs. 5 and 8 show, there was a statistically significant effect of speaker style, such that preference for the literal target increased in response to the nonliteral speaker during the late time window. This contrasts to the preference for the literal target in response to the literal speaker, which remained relatively constant between the early and late stages. Finally, unlike in the early window, there was not a statistically significant effect of trial order.

All data and code are available at https://osf.io/q7f2r/.

## Discussion

This study investigated the influence of speaker-specific style on semantic disambiguation. In line with the literature on speaker-specific adaptation on other domains of language, we hypothesised that when interpreting polysemous words with a literal and a nonliteral sense, habituated addressees would experience a greater pull towards a nonliteral sense in response to a nonliteral speaker than in response to a literal speaker, before ultimately resolving to the literal target.

As hypothesised, our offline data showed that the vast majority of responses resolved to the literal target, regardless of speaker style. Our online measures supported the interference hypothesis, though more subtly than hypothesised. Against our prediction of slower resolution in the nonliteral style condition, there was no effect of speaker style on reaction times. Likewise, there was no effect of speaker style on proportion of looks during the early time window (during articulation of the noun), contrary to our prediction of fewer looks to the literal target in response to the nonliteral speaker.

However, in the late time window (the post-noun period), preference for the literal target significantly increased in response the nonliteral speaker. This finding is consistent with what is known in the ambiguity resolution literature as the *subordinate bias effect* (e.g., Brocher et al., 2016; Rodd, 2020). In this line of research, when an ambiguous word is presented in a preceding context that strongly biases its subordinate meaning, there is a delay in the recognition of the ambiguous word (compared to a neutral control) caused by the initial processing of its dominant meaning. We suggest that in our experiment, the increased proportion of looks to the literal target in response to the nonliteral speaker at later stages reflects this earlier processing of the dominant/literal meanings. Since we did not rely on sentential context (to create a more direct bias) in our design but purely on the extralinguistic context created by speaker style, our findings support our initial hypothesis that speaker style would influence the semantic disambiguation process.

We interpret the preponderance of literal-target resolution in the response-type results as a straightforward effect of meaning dominance on ultimate referential commitment, overriding any potential effect of speaker style. All of our items were high-frequency nouns, normed as having a literal bias in the pretest. This bias is borne out in participants’ ultimate referential choices.

The finding that participants resolved to the literal target^3^ equally quickly regardless of speaker style was somewhat surprising. It suggests that any consideration of the nonliteral target as the intended referent did not incur an online time penalty. As with the response data, we attribute this to meaning dominance effects overriding potentially more subtle speaker style effects. Future work that minimises dominance between interpretations might enable any speaker style effects to manifest more clearly. For example, using stimuli that bias towards the nonliteral such as ‘link’, ‘figure’, and ‘fight’ (i.e., the items that did not go forward from the pretest) may pattern differently with respect to speaker style effects. Indeed, an exploratory post-hoc split-half analysis which divided the 32 experimental items into high- and low-metaphoricity (based on the ratings shown in Fig. 1) revealed a trend for the high-metaphoricity items to yield a stronger preference for the literal target in the late time window, compared to the low-metaphoricity items. This suggests that these items were driving the overall effect in the eye movement findings reported above. Other paradigms to consider in future work include a between-items design that uses only a literal target (alongside distractors) for some trials and only a nonliteral target in other trials, intended to increase clicks to the nonliteral targets, and, thus, a greater chance to observe a speaker style effect. Another option would be to use a speech comprehension paradigm in which a sentence like “the woman spent hours in front of the mirror making a stylish bun” might feasibly be easier to interpret if spoken by a nonliteral than literal speaker^4^.

The analysis of early- and late-stage eye movements addresses the question of whether speaker identity affects this literal bias during processing. In response to the literal speaker, addressees showed a reduced preference for the literal target during the late, post-noun period. At noun onset, they are initially guided by the dominance of literal meaning. Then having confidently resolved reference to the literal target during the noun, their interest in it drops away as they survey the other images on screen. Crucially, we do not see a similar reduction in attention during the late time window in response to the nonliteral speaker. The continued processing in the nonliteral condition reflects a late checking or confirmation of the dominant target, which we interpret as a result of an earlier indeterminacy. Although our data does not significantly evidence this uncertainty or equivocality in the earlier time window, the trend for weaker target preference in the nonliteral condition would support such an interpretation. This late-stage re-focusing reflects the point at which dominance effects activate greater attention on the literal target after an earlier stage when both meanings were accessible.

Thus, our data reveals a subtle effect of speaker style, manifest as a late-arriving, reduced preference for the dominant referent in response to the literal speaker, and increased preference for the dominant referent in response to the nonliteral speaker. We take this to be a result of earlier, more reliable cues to literal meaning in the literal speaker condition, and to dominance cues coming online later in the nonliteral speaker condition. In our task, the visual context presents the *possibility* of a nonliteral meaning, with a nonliteral speaker triggering an early, fleeting interference with dominance cues, manifest as a delayed preference for the dominant meaning. Note that we elicited this effect after subjecting participants to a fairly lengthy implicit exposure phase followed by a more explicit association task. We do not anticipate that speaker style effects would have been manifest using less explicit training. On the other hand, if the training materials had included the same type of metaphorical polysemes that appeared in the test materials rather than the more general conceptual metaphors that were used, we may have seen stronger effects of speaker style such as those originally hypothesized (though such an approach might have introduced strategic responding by the participants).

We also analysed the effect of trial order. Eye movement data showed an increasing preference for the literal target over the course of the experiment, with a stronger preference for nonliteral trials in earlier trials. These results shed light on how expectations about speaker intentions are updated. The stronger preference for nonliteral targets in earlier trials and increasing preference for literal ones over the course of the experiment suggest a gradual weakening of the habituation effect that was set up in the training phase. Although addressees generalised from the nondominant meanings used by the speaker during the training phase to new nonliteral referents, consideration of the nonliteral target in both style conditions was stronger closer in time to the training phase. Then, regardless of speaker, addressees increasingly relied on dominance cues (manifest as preference for the literal target) as the experiment proceeded in the absence of any updated evidence that the speaker is nonliteral. This gradual decay may be driven by priming effects. It does not appear to be the case that nonliteral meanings are directly activated in response to merely seeing a nonliteral speaker (with the same holding for the literal speaker): there were no anticipatory eye movements during the speaker preview region. Instead, once the speaker had started their utterance, this, coupled with the option of a nonliteral target onscreen, seemed to lead participants to remain open to the possibility of a nonliteral target, at least in the early part of the experiment. This pattern may be driven by a learning effect whereby addressees came to realise that there was always a fit for the dominant meaning of the polysemous expression and searched it out as they progressed through the items, disregarding the nonliteral target to complete each trial.

Our findings align with the speaker-model of processing proposed by Cai et al., (2017). Under this account, a model of the speaker is established during exposure and is used to guide meaning access for subsequent speech, for example to boost speaker-congruent meanings. As Cai et al. propose (2017, p. 76), a speaker model “allows listeners to use a variety of linguistic and paralinguistic cues to build up a representation of the speaker that contains information about, for example, their age, gender, voice identity (e.g., Belin et al., 2004) or social background (Sumner et al., 2014). Based on our findings, we add speech style to Cai et al.’s list of speaker-based cues in speech interpretation.

A single entry model of polyseme processing states that there is a common core representation that is activated, rather than a ranked list of likely senses (Klepousniotou et al., 2008; Nunberg, 1979). A later stage of optional specification can then follow, necessitated to a greater or lesser degree by the amount of contextual weighting and the requirements of the task. Our results support this hypothesis, with the literal specification coming earlier in response to the literal speaker and later in response to the nonliteral speaker. Meaning dominance has the ultimate influence on disambiguation (Klepousniotou et al., 2008), yet the between-condition differences during the post-noun period suggest that speaker-related factors have an influence prior to referential commitment.

The current study used a range of online and offline measures to investigate the role of speaker style in semantic processing. In comparison to previous work on semantic disambiguation, our stimuli did not include a biasing sentential context. In our paradigm, the *speaker* is the preceding (as well as co-occurring) context that modulates meaning expectations. The fact that the polysemous nouns did not appear in a literal or nonliteral sentential context means that biasing information could not be carried by the meaning of the sentence. Instead, the temporary bias away from the literal meaning came from the speaker, or more specifically from the addressee’s expectations of that speaker’s preferred meaning, to be used throughout the subsequent interaction. In line with the large body of work on the use of context in semantic disambiguation (Binder & Morris, 1995; Duffy et al., 1988, 2001) our findings underscore the fact that context is key. We extend this work by providing evidence that speakers themselves help shape context. When ambiguous words occur devoid of sentential context, the speaker is used as a determinant of context (Cai et al., 2017). Thus, our findings extend the literature on partner-specific effects to semantic processing. In line with results from other domains of language processing, we have shown that listeners can make inferences about a speaker and use that information to constrain their interpretation of the intended meanings of ambiguous words. In doing so, listeners integrate multiple cues to meaning, including those of a social and experiential nature.

Our results characterise speaker style effects as subtle, subject to decay, and weaker than meaning dominance. Addressees use speaker style cues in decision making, but these cues are transient and ultimately insufficient for them to consistently override meaning dominance cues. However, it is striking that even against the strength of the ultimate preference for literal targets, the effect of speaker style is nevertheless evident. In spite of the inherent bias to the literal interpretation (due to the intentional dominance of literal meanings in our stimuli), speaker style acted as an additional cue to meaning during processing. Our results provide evidence that speaker style is a contextual determinant in semantic disambiguation using polysemous words. Addressees can make inferences about a particular speaker and then use that information to constrain their interpretation of the intended meanings of the words produced by that speaker. Our findings extend the literature on partner-specific effects to the domain of semantic processing. Furthermore, our results support theoretical accounts proposing that semantic comprehension involves rapid integration of multiple cues including those of a social nature. Ongoing work in experimental pragmatics shows that speakers have considerable influence on language processing. The evidence that we have presented illustrates the influence of specific speakers’ identity on meaning interpretation, broadening the conceptualisation of speaker meaning.

## Footnotes

1 The current study examines the role that specific speakers play in meaning retrieval within a discourse context; a role highlighted by the very term ‘speaker meaning’.

2 Although we focus here on metaphoricity as an example of nonliterality, this choice is somewhat arbitrary and other styles could have been chosen, e.g., prosaic, concise, hyperbolic, sarcastic, etc. The aim of the study is to investigate the effect of speaker style on semantic processing, rather than to comment on mechanisms of nonliteral language processing.

3 A further analysis of response times to all trials (recall that the analysis reported above included only those trials that were resolved to the literal target) revealed that the few responses to the nonliteral target were slower than those to the literal target (untransformed reaction times: LT M = 2073 ms, SD = 844; NLT M = 2786 ms, SD = 1189, estimate 0.10, SE 0.04, df 32.42, t = 2.24, *p* < .05). This suggests that participants require more time to settle on a nondominant interpretation, presumably due to their relatively lower frequency.

4 We thank an anonymous reviewer of an earlier version of this manuscript for this suggestion.
